# Supplementary material for: Three-dimensional printing of photonic colloidal glasses into objects with isotropic structural color
Source: Nat Commun. 2022 Jul 29;13:4397. doi: 10.1038/s41467-022-32060-2 (PMC9338281; doi:10.1038/s41467-022-32060-2)
Supplement: Supplementary file 1 — Supplementary Information [file 41467_2022_32060_MOESM1_ESM.pdf]

Supplementary Information for the paper

"**Three-dimensional Printing of Photonic Colloidal Glasses into Objects with Isotropic Structural Color**"

Ahmet F. Demirörs[*,1], Erik Poloni[1,†], Maddalena Chiesa[1], Fabio L. Bargardi[1], Marco R. Binelli[1], Wilhelm Woigk[1], Lucas D. C. de Castro[1,2,‡], Nicole Kleger[1], Fergal Coulter[1], Alba Sicher[3], Henning Galinski[4], Frank Scheffold[5] and André R. Studart[*,1]

[1] Complex Materials, Department of Materials, ETH Zurich, 8093 Zurich, Switzerland

[2] Federal University of São Carlos, Department of Materials Engineering, São Carlos, SP, Brazil

[3] Living and Soft Materials, Department of Materials, ETH Zurich, 8093 Zurich, Switzerland

[4] Nanometallurgy, Department of Materials, ETH Zurich, 8093 Zurich, Switzerland

[5] Soft Matter and Photonics, Department of Physics, University of Fribourg, 1700 Fribourg, Switzerland

[†]Currently at: High Enthalpy Flow Diagnostics Group, Institute of Space Systems, University of Stuttgart, 70569 Stuttgart, Germany

[‡]Currently at: São Carlos Institute of Physics, University of São Paulo, 13566-590 São Carlos, SP, Brazil

E-mail: ahmet.demiroers@mat.ethz.ch and andre.studart@mat.ethz.ch

Supplementary Notes

Supplementary Note 1. Direct Ink Writing of distortion-free complex-shaped objects:

Guidelines for direct ink writing have been proposed in terms of the yield stress and storage modulus levels needed to prevent capillary- and gravity-driven distortions of grid-like structures. [44, 45]

On the basis of simple beam theory, we first estimate the storage modulus (G') levels required to print distortion-free grid-like architectures (Figure 2a). In this estimation, the span length (L) of a filament hanging in air between two underlying print lines is considered an important geometrical parameter of the grid. Earlier work has shown that filament sagging is minimized if the storage modulus of the ink (G') satisfies the following condition: $G' \geq 1.4wD(L/D)^4$, where D is the filament diameter and w is the specific weight of the ink ($\rho g/4$). [44] Taking typical values for the specific weight of the colloidal ink (3213 $kg \cdot m^{-2} \cdot s^{-2}$) and for the filament diameter (0.58 mm), the measured storage modulus of 18.8 kPa (Figure 2a) should allow for the fabrication of grids with specific span length up to $L^* = 9.2D$ without significant sagging.

To provide guidelines for the yield stress of the ink (τ_y), we consider the capillary stress (ΔP) that develops across a curved surface: $\Delta P = \gamma/r$, where γ is the surface tension and r is the radius of curvature. To prevent capillary-induced distortion, the yield stress of the ink should be larger than the capillary stress: $\tau_y \geq \Delta P$. This leads to the following simple estimation for the minimum radius of curvature for a distortion-free object: $r = \gamma/\tau_y$. Assuming a surface tension of 0.019 N/m for our colloidal inks,[45] we predict that the yield stress of our ink (441 Pa) should enable printing of objects with specific radii of curvature down to 43 μm without capillary-induced distortion.

Supplementary Note 2. Thermal gravimetrical analysis of the ink and the ink constituents:

To understand and interpreted the different parts of the TGA curve shown in the main text (Figure 3b), we have also measured the thermally induced weight loss of the individual constituents of the 3D printing ink. TGA was conducted for the PEO-PPO-PEO copolymer (Pluronic F108), silica particles, carbon black (CB) and for the 3D printed green body after room-temperature drying (Supplementary Figure 1). As expected, the silica and CB particles show only relatively low mass losses under the investigated temperature range. The 5% weight loss observed for the silica particles until 400 °C is likely due to the evaporation of physically and chemically bound water or possibly the decomposition of organics added during synthesis (Supplementary Figure 1b). CB starts to lose weight only above 700 °C, when carbon is expected to be oxidized (Supplementary Figure 1c).

Interestingly, the PEO-PPO-PEO copolymer exhibits a strong weight drop at 300°C and loses almost all its mass at 400 °C (Supplementary Figure 1a). By comparing the TGA curve obtained for the whole ink with the measurements of the individual components (Supplementary Figure 1e), we find that the ink weight loss at 200°C is probably due to the decomposition of the PEO-PPO-PEO copolymer at this temperature. Indeed, the weight percentage of this loss corresponds well with the weight percentage of the copolymer in the ink. Upon removal of the copolymer, the particle assembly densifies and the structural color emerges.

The reduction in decomposition temperature from 300°C to 200°C observed when the copolymer is incorporated into the ink is probably related to the different configuration of the molecules in the aqueous suspension. The PEO-PPO-PEO copolymer is a hydrophilic molecule that swells in an aqueous solution at ambient temperature. Moreover, these molecules are expected to adsorb on the surface of the silica and carbon black particles present in the ink. These factors likely reduce the thermal stability of the copolymer, thus lowering the decomposition temperature.

The slight mass gain observed during the thermogravimetric analysis of the ink (Figure 3b) was confirmed by repeating the TGA experiment (Supplementary Figure 1d). This small peak is likely due to the uptake/absorbance of oxygen as a result of oxidation of the dried material. Such oxidation occurs right before the copolymer starts to decompose at this temperature.

To gain further insights into the thermal decomposition of the copolymer alone, an additional TGA measurement was performed under the same heat treatment applied to the printed objects (Supplementary Figure 1f). In this experiment, the temperature is held at 200°C after an initial quick heating of the sample. The results show that the copolymer loses ~90% of its weight after a 1-hour heat treatment at 200°C. This confirms that the emergence of color during heat treatment results from the densification that takes place upon the removal of water and copolymer from the printed assembly.

Supplementary Note 3. Linear shrinkage estimation:

We estimated the shrinkage by two methods one was using simple image analyses on the SEM micrographs. The second was considering the material properties of the ink contents and assuming the whole water removal. In the image analyses method, the particle numbers under the same area under SEM images were counted and compared for RT dried and 200 °C heated samples, see Supplementary Figure 1. By comparing such particles density over the area, we estimated a linear shrinkage of 14%. The second estimate was performed by comparing the densities and volumes of the ingredients of the ink formulation. We compared the initial volume of the ink to the volume of the ink considering full removal of water. This resulted in a linear shrinkage estimate of 16 %.

Supplementary Note 4. Details of the SAXS detector:

The SAXS data were collected using detectors based on hybrid pixel technology. Unlike CCD detectors, large surface hybrid pixel detectors are composed of side-butted chips, generating discontinuous arrays. This discontinuity is the reason for the vertical lines observed in SAXS patterns shown in Figure 3d and Supplementary Figure 2a. Although the data could be 2D interpolated for aesthetic purposes, it was chosen here to keep the original and unbiased 2D data. Hybrid pixel detectors have advantages over CCD technology with superior Point Spread Function (PSF) of 1 pixel against 2-3 pixels for CCDs and with absence of read out noise over 9 decades of dynamic range against a maximum of 4 decades of dynamic range and easy saturation of CCDs.

Supplementary Note 5. Influence of the 3-roll milling step on the colloid volume fraction in the ink:

We have used a 3-roll mill to homogenize the ink and break down particle aggregates that could cause nozzle clogging during DIW printing. During the 3-roll milling process, the ink is exposed to air and it is spread on the high-surface-area rolling cylinders. This leads to water evaporation and lowers the water content of the ink. Therefore, it is not easy to control the water content of the ink during this homogenization step. As a result of this evaporation effect, the final solid volume fraction and the

distance between colloids after the 3D printing and heat treatment steps may not match closely the predicted values. This affects directly the reflectance of the printed object as shown in earlier work[1].

Supplementary Note 6. Reflectance of printed structures for different detection angles:

The reflectance of 3D printed structures was measured as a function of the observation angle by illuminating the sample with collimated white light from a DH-2000-BAL Deuterium-Halogen Lightsource (Ocean Optics) using an optical fiber. The angle between the incident light and the normal to the sample surface was kept at 0°. The size of the illuminated spot on the sample was ~3 mm in diameter. A detector mounted on a rotating arm was used to measure the reflectance of the sample at different angles, ranging from 15° to 65° (θ) from the normal direction of incidence. The detector consisted of an aperture, a collecting lens and a second optical fiber connected to a QE-Pro High Performance Spectrometer (Ocean Optics). At the start of the measurement, a reference spectrum was measured leaving the sample holder empty. Dark noise was measured leaving the shutter blocking the incident light closed. The reflectance was calculated as the ratio between the reflected intensity minus dark noise and the reference spectrum minus dark noise.

Supplementary Note 7. Resolution of the DIW 3D printing method:

The resolution of the DIW method usually lies around 100 µm, but can be extended down to a few microns by printing the ink in a liquid at high extrusion pressures[2]. Here, we used a 410 µm nozzle to achieve sufficient resolution without causing clogging during the printing process. DIW of particle-containing inks below 100 µm resolution is challenging, since clogging might become an issue for nozzles below this size. This originates from the fact that our ink has a high concentration of particles. Therefore, any particle aggregates in the suspension have to be disintegrated within the ink before printing. To this end, the particle suspensions were subjected to a 3-roll milling process, which reproducibly led to homogenous inks that can be flawlessly ejected from 410 µm nozzles. In principle, ball-milling the suspensions over long hours may help to further remove aggregates and possibly improve the resolution of the DIW process. Alternatively, the resolution can be enhanced by developing inks that can be printed using stereolithography techniques[3].

Supplementary Figures

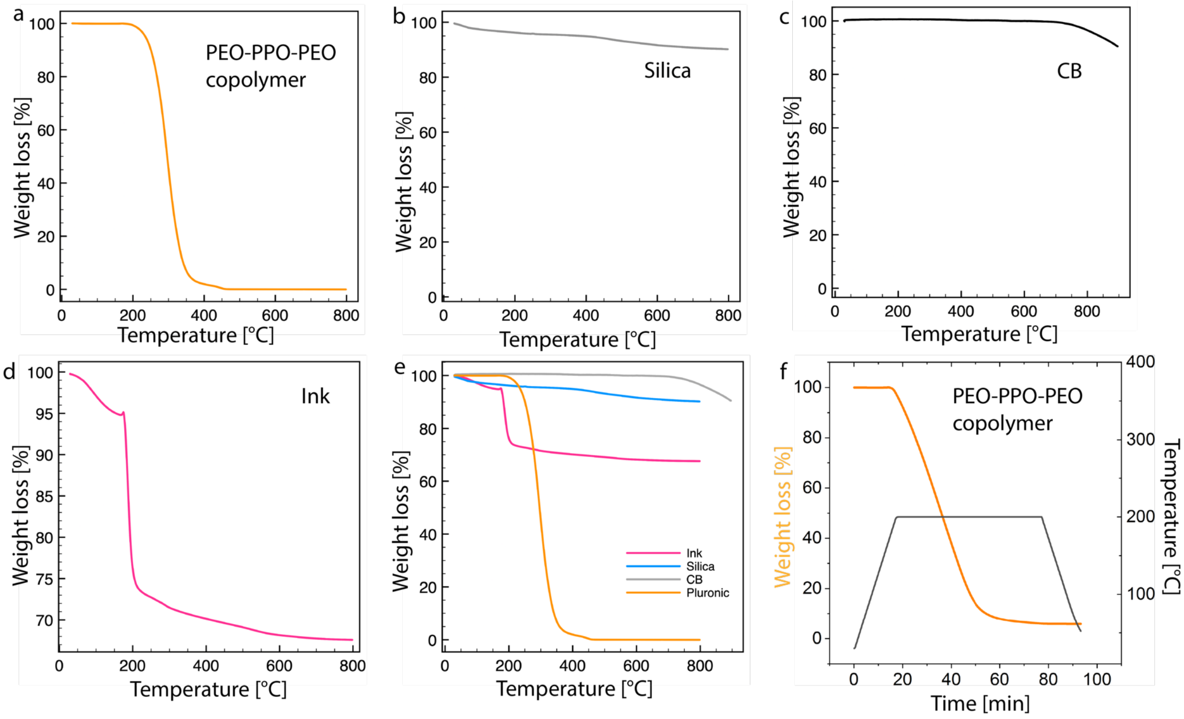

Supplementary Figure 1. Thermal gravimetric analysis (TGA) of the ink components. TGA curves taken from 30 to 800°C of (a) PEO-PPO-PEO copolymer (Pluronic F108), (b) 250-nm silica particles, (c) carbon black (CB) and (d) the produced ink. (e) Combined plot of curves shown in (a-d). (f) TGA of the PEO-PPO-PEO copolymer alone under isothermal conditions at 200°C. Here, the sample was heated at a speed of 10°/min up to 200 °C and the temperature was held at 200°C for 1 h to simulate our drying experiments. Source data for plots are provided as a Source Data file.

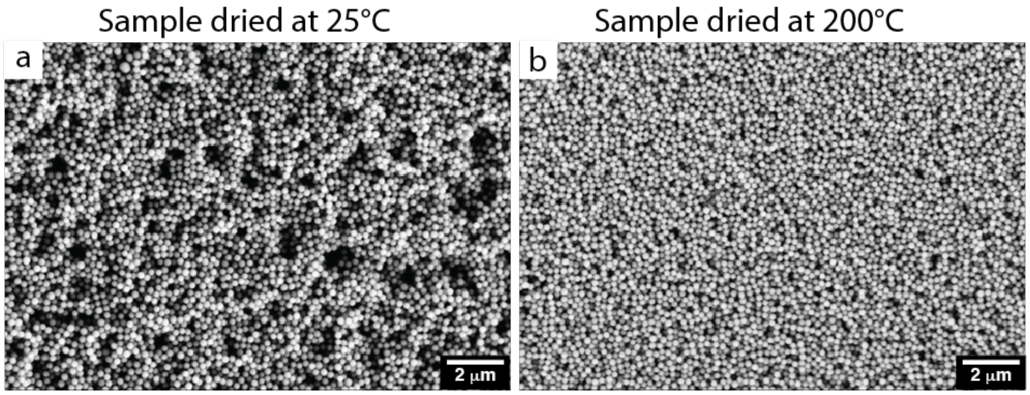

Supplementary Figure 2. Microstructure of colloidal glass formed upon drying at 25 °C and heating 200 °C. (a,b) SEM images of printed samples dried at (a) 25 °C and heat treated at (b) 200 °C. Specimens were printed from inks containing 250 nm silica particles. The number of particles in the

images increases from 1834 particles in (a) to 2758 particles in (b). Based on these particle densities, we estimate a linear shrinkage of ~14% during the heat treatment at 200 ºC.

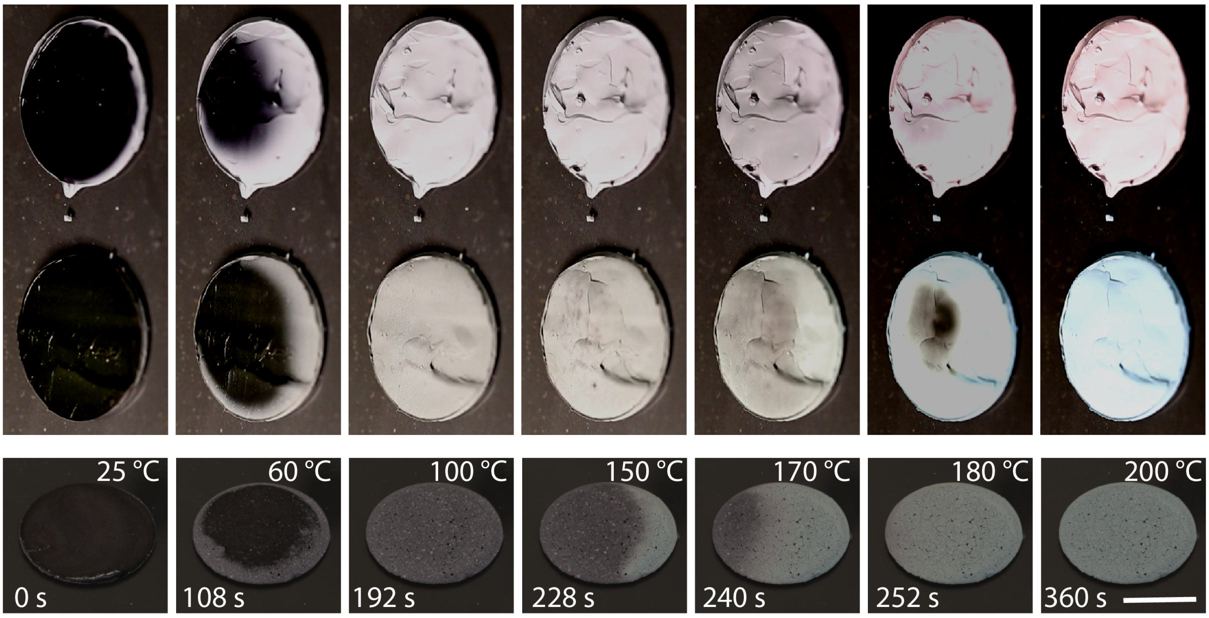

Supplementary Figure 3. Evolution of the color of the silica structure during heating. The photographs depict a disc-shaped sample made from an ink containing 250 nm silica particles. The sample was subjected to quick heating to demonstrate the emergence of color as the temperature increases. The first color change of the sample is observed when the water starts to evaporate upon heating up to 100°C. Removal of water changes the color from black to gray. At 150°C the PEO-PPO-PEO copolymer (Pluronic F108) starts to melt and disintegrate. As a result of water and copolymer removal from the structure, the particles rearrange and densify to form a colloidal glass. Green structural color emerges at this stage. At the higher temperature of 200°C, the colloidal glass is consolidated all over the sample. Scale bar is 1 cm.

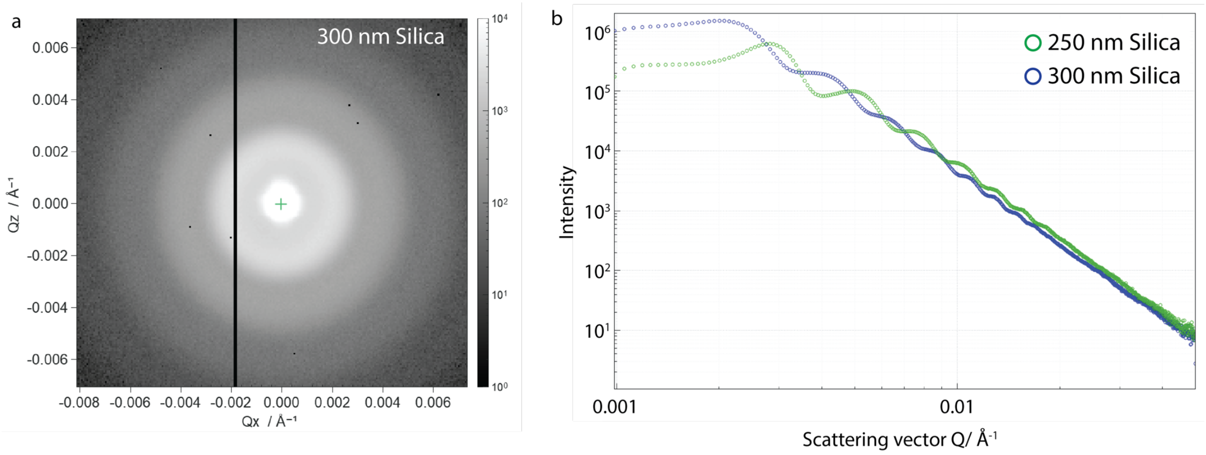

Supplementary Figure 4. SAXS measurement on an object printed from an ink containing 300 nm silica particles. (a) SAXS pattern and (b) azimuthal X-Ray intensity as a function of scattering vector (Q) obtained after heating at 200 °C, shown for 250 and 300 nm particle containing inks. From full width at half maximum of the intensity peak (ΔQ), we estimate the range of spatial order to lie around 550 nm for 300 nm particles.

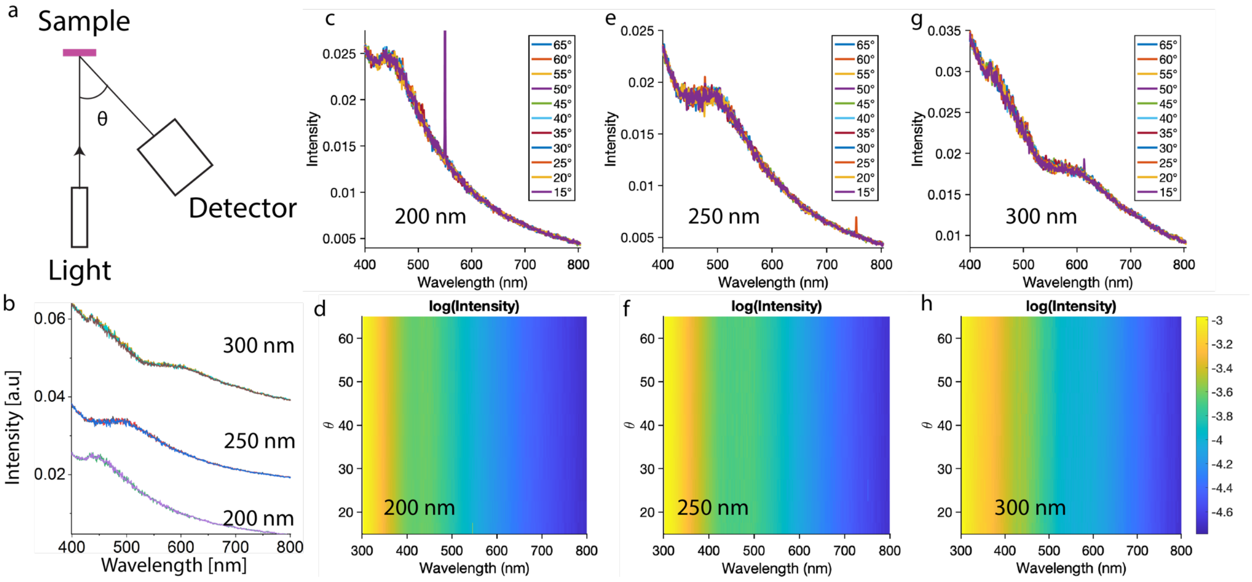

Supplementary Figure 5. Reflection spectra of printed photonic glass structures as a function of the observation angle. (a) Sketch of the used set up. Angle reported in the plots is the angle θ shown in the sketch. (b) Three spectra obtained for angles θ =15, 40 and 65° were overlaid to demonstrate the angle independence of the color of samples prepared with different silica particle sizes. (c,e,g) Spectra of the photonic glass assembled from (c) 200 nm, (e) 250 nm and (g) 300 nm particles at angles θ varying between 15 and 65°. (d,f,h) Heat maps displaying the effect of the angle (θ) and the wavelength on the log of reflection intensity for samples prepared with (d) 200 nm, (f) 250 nm, and (h) 300 nm silica particles.

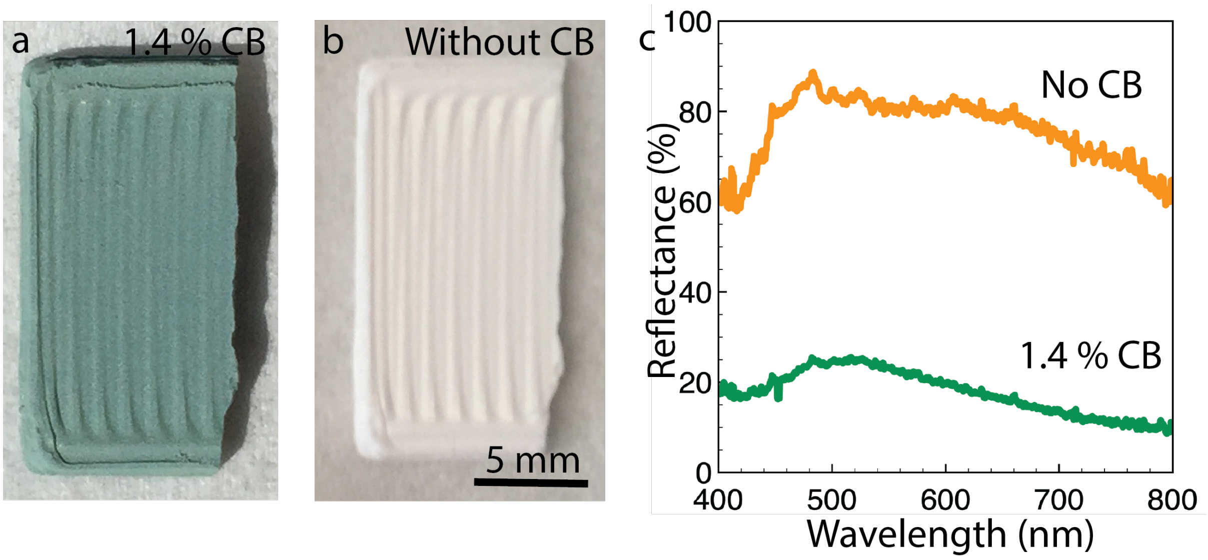

Supplementary Figure 6. **Comparison of printed samples with and without the addition of carbon black (CB).** (a,b) Photographs of a printed sample prepared with 250 nm sized particles containing (a) 1.4 % CB and (b) no CB. (c) Reflectance spectra of 3D printed samples with and without CB. The presence of carbon black in the 3D printed sample allows for the emergence of the green color. Upon heat treatment of this same sample at 850°C for 3 hours, the CB is removed and the color turns to white with stronger reflectance. This suggests that the CB inhibits multiple scattering events and thus saturates the color.

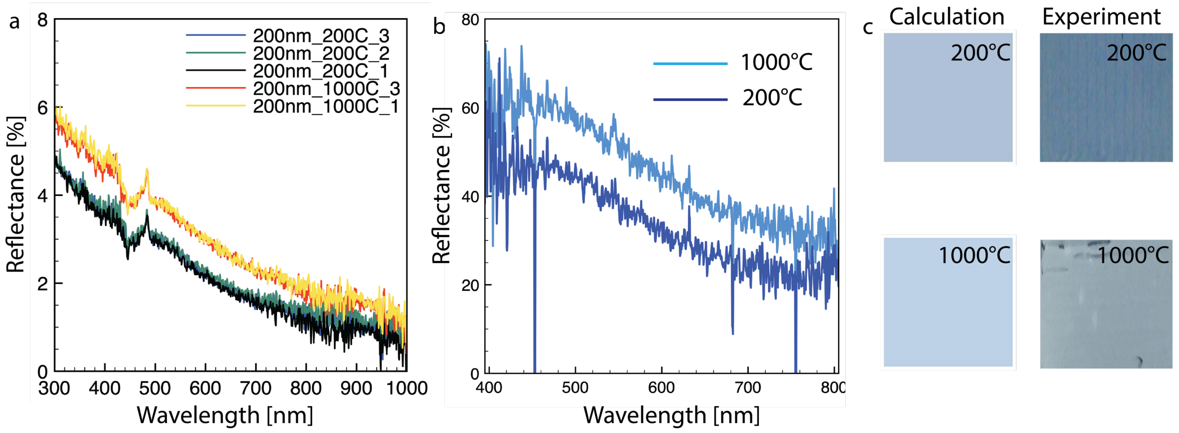

Supplementary Figure 7. Reflectance measurements of samples prepared with 200 nm silica particles dried at 200°C and 1000°C. (a) Near incidence angle reflectance of the samples measured up to 3 times. (b) Total reflectance of the two samples performed with an integrating sphere. (c) Colors calculated from the total reflectance plots in (b) are compared with the photographs of the samples. Source data for (b) are provided as a Source Data file.

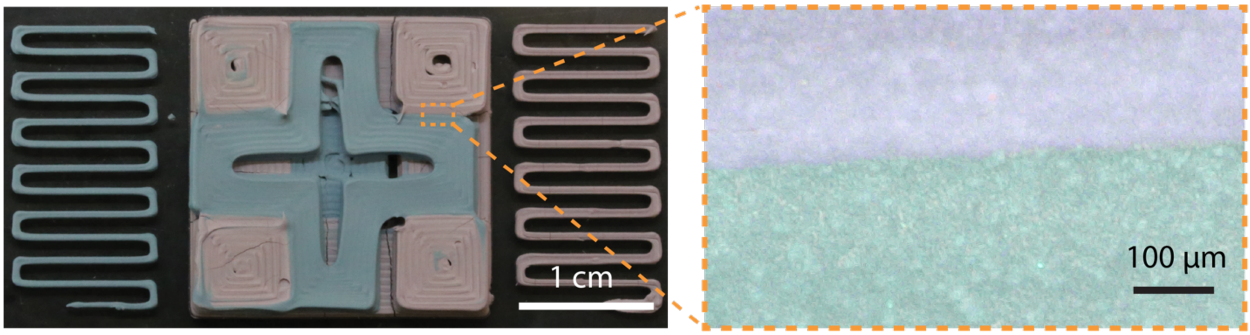

Supplementary Figure 8. Interface between the colored domains of a multimaterial printed object. The reflection optical microscopy image (on the right) of the interface between the two differently colored domains demonstrates the sharpness of the printing lines. Such a sharp interface was obtained using the same nozzle diameter of 580 µm, which indicates that the resolution of the 3D printing process is not lowered when multiple inks are used.

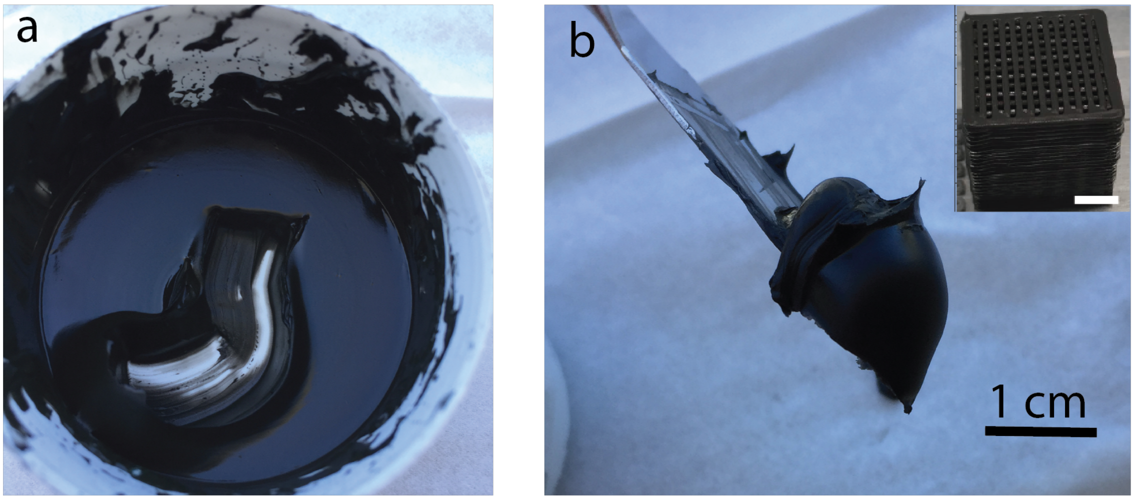

Supplementary Figure 9. **Photographs of the ink containing carbon black.** (a) Highly homogenous ink obtained after the 3-roll milling process. (b) Ink manipulated with a spatula. The inset shows the 3D printed grid before the heat treatment. Scale bar in the inset is 5 mm.

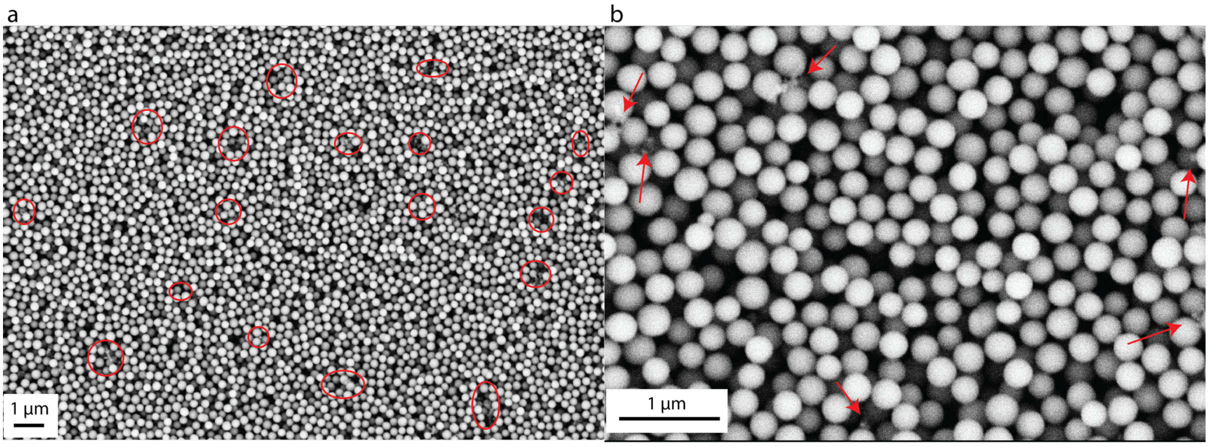

Supplementary Figure 10. SEM micrographs of the printed structure after the heat treatment. (a,b) Images obtained at the magnifications of (a) 14k and (b) 48k. The homogenous distribution of the carbon black particles through the ink is illustrated using the red marks. Since the CB shows an average size of 7 nm, most of the particles are not visible in these images.

Supplementary Table

Supplementary Table 1. Average hydrodynamic diameter and polydispersity of the silica particles measured by Dynamic Light Scattering (DSL). *

AngstromSphere reported size [nm]	Light Scattering size [nm]	Polydispersity [%]
200	234	2
250	256	2
300	311	6

* The particle size analyses (DLS) were performed with a Malvern Zetasizer Nano ZS instrument. The power of DLS to quantify small polydispersities is limited, but for a measured DLS-polydispersity << 10% it sets an upper bound of approximately 10% in agreement with the supplier specification[4]. Source data for measurements are provided as a Source Data file.

Supplementary References

1. Hwang, V. *et al.* Designing angle-independent structural colors using Monte Carlo simulations of multiple scattering. *Proc Natl Acad Sci U A* **118**, (2021).
2. García-Santamaría, F. *et al.* A Germanium Inverse Woodpile Structure with a Large Photonic Band Gap. *Adv. Mater.* **19**, 1567–1570 (2007).
3. Brown, T. E. *et al.* Voxel-Scale Conversion Mapping Informs Intrinsic Resolution in Stereolithographic Additive Manufacturing. *ACS Appl. Polym. Mater.* **3**, 290–298 (2021).
4. Pusey, P. N. & van Megen, W. Detection of small polydispersities by photon correlation spectroscopy. *J. Chem. Phys.* **80**, 3513–3520 (1984).
